# Cerebral Blood Flow and Other Predictors of Responsiveness to Erenumab and Fremanezumab in Migraine—A Real-Life Study

**DOI:** 10.3389/fneur.2022.895476

**Published:** 2022-05-17

**Authors:** Magdalena Nowaczewska, Marcin Straburzyński, Marta Waliszewska-Prosół, Grzegorz Meder, Joanna Janiak-Kiszka, Wojciech Kaźmierczak

**Affiliations:** ^1^Athleticomed—Pain & Sport Injury Centre With Headache & Migraine Treatment Division, Bydgoszcz, Poland; ^2^Department of Otolaryngology, Head and Neck Surgery, and Laryngological Oncology, Ludwik, Rydygier Collegium Medicum in Bydgoszcz, Nicolaus Copernicus University in Toruń, Bydgoszcz, Poland; ^3^Headache Clinic—Terapia Neurologiczna “Samodzielni, ” Warsaw, Poland; ^4^Department of Neurology, Wroclaw Medical University, Wrocław, Poland; ^5^Department of Interventional Radiology, Jan Biziel University Hospital No. 2, Bydgoszcz, Poland; ^6^Department of Human Physiology, Collegium Medicum in Bydgoszcz, Nicolaus Copernicus University in Toruń, Bydgoszcz, Poland

**Keywords:** CGRP, erenumab, fremanezumab, cerebral blood flow, migraine, outcome, headache, transcranial Doppler

## Abstract

**Introduction:**

Monoclonal antibodies (mAbs) showed efficacy in migraine prevention. The aim of this study was to check if baseline clinical parameters and cerebral blood flow (CBF) measured by transcranial Doppler (TCD) may help predict mAbs efficacy.

**Methods:**

Electronic charts of migraineurs treated with erenumab or fremanezumab, with baseline TCD evaluations were collected, including data on migraine type, pain localization, monthly migraine days (MMD), medication overuse headache (MOH), mean blood flow velocity (Vm), and pulsatility index (PI) in cerebral arteries.

**Results:**

A total of 123 patients were enrolled, mean age 38, 75 years, 87 with chronic migraine, 61 with MOH, 72 were good responders (GR), and reported ≥50% reduction in MMD, 43 ≥75% reduction in MMD. Baseline Vm values in MCAs were significantly lower in GR as compared with non-responders. MAbs responsiveness ≥50% was positively associated with unilateral pain localization (OR: 6.53, 95% CI: 2.01–23.93; *p* = 0.003) and HIT-6 score (OR: 1.14, 95% CI: 1.01–1.30; *p* = 0.036) whereas negatively associated with Vm in right MCA (OR: 0.96, 95% CI: 0.92–0.99; *p* = 0.012), and having no relatives with migraine (OR: 0.40, 95% CI: 0.16–0.95; *p* = 0.040).

**Conclusions:**

Baseline Vm in MCA is lower in mAbs GR as compared with non-responders which may reflect increased secretion of CGRP with further vasodilation in GR. Simple clinical features and baseline CBF in anterior circulation might help to predict the patient's responsiveness.

## Introduction

Calcitonin gene-related peptide (CGRP) plays an important role in the pathophysiology of migraine and anti-CGRP monoclonal antibodies (mAbs) have been developed and shown efficacy in the prevention of migraine attacks (1). However, we are not able to select which patient is a good candidate to treat by predicting efficacy response. Transcranial Doppler ultrasonography (TCD) makes it possible to assess blood flow in the intracranial arteries non-invasively. TCD parameters are influenced both by changes in cerebral vessels diameters and CBF, moreover, CBF correlates with the flow velocity in these vessels (2). On the other hand, it is known that CGRP may dilate cerebral and extracranial arteries and mAbs were reported to inhibit CGRP-induced vasodilatory responses in human arteries (3–7). Our previous pilot study found that baseline mean flow velocity (Vm) in cerebral arteries measured by TCD is lower in mAbs good responders (GR) as compared with non-responders, which may predict treatment efficacy (8). The aim of this study was to replicate this finding in a larger cohort as well as to identify any other predictors of mAbs efficacy.

## Materials and Methods

This retrospective observational cohort study involved consecutive migraineurs treated at our Headache Center and receiving the clinical indication for migraine prevention with mAbs (Aimovig, Novartis Europharm Limited or Ajovy, Teva Pharmaceuticals).

Patients were included in this cohort analysis if they had a diagnosis of migraine with or without aura according to the International Classification of Headache Disorders (ICHD-3), at least 4 migraine days for a month, were aged between 18 and 70 years, received treatment with mAbs for at least 3 months, had baseline TCD examination performed before starting the treatment and had to follow up visit after starting the treatment (9). Patients were excluded if they had inadequate temporal windows, stenosis of the intracranial arteries, hemodynamically significant stenosis of the internal carotid arteries, atrial fibrillation, cardiovascular disease and other severe somatic or psychiatric disorders, treatment with migraine prophylactic drugs, or other drugs which might influence CBF on the day of TCD assessment. Patients reporting migraine headaches or taking triptans on the day of TCD assessment were excluded from the study.

Data were extracted from 2019 through November 2021 from the electronic medical database. All patients underwent the clinical and TCD evaluations at baseline before initiating mAbs treatment. In our center, every headache patient undergoes routine TCD evaluation on baseline visit. Data on migraine onset age, migraine type, pain location, type of pain, presence of additional migraine symptoms (nausea, vomiting, photo, and phonophobia), monthly migraine days (MMD), monthly headache days (MHD), acute medication days (AMD), type of acute medication used, headache intensity using a numerical scale (numeric rating scale, NRS), headache burden using the Headache Impact Scale (HIT-6), number of previous preventive classes failures (antidepressants, antiepileptic, beta-blockers or antihypertensive drugs, botulinum toxine), responsiveness to triptans, and onabotulinumtoxin A, family history of migraine, comorbidities, and concomitant medications, were collected. The pain was considered unilaterally fixed (side-locked) if it occurred on the same side of the head for more than 90% of migraine attacks, unilateral variable if unilateral but changed the side of the head between attacks or during the attack or bilateral in all the other cases. Patients were also classified as suffering from episodic (EM) or chronic migraine (CM) according to the ICHD-3 (9). Patients with all degrees of medication overuse headache (MOH) defined according to the ICDH-3 were included (9). Treatment efficacy was assessed based on the patients' last month of receiving mAbs according to the patient's headache diary. Patients were evaluated after 3 months of treatment.

Based on treatment efficacy we divided patients into two groups: good responders and non-responders. Patients, who reported a good effect of treatment (≥50% reduction in MMD), were defined as good responders. The remaining patients were defined as non-responders. Then we compared TCD parameters and clinical data between those two groups. We also defined the super-responders group (≥75% reduction in MMD).

Our study was approved by the Local Ethics Committee of the Ludwik Rydygier Collegium Medicum in Bydgoszcz. Specific written consent was not required for this retrospective study.

TCD examinations were performed with Nicolet Sonara transcranial Doppler system (Viasys Healthcare) and a 2 MHz probe. The examination was performed in a quiet room with the subjects lying in a comfortable supine position, after 10 min rest, using a standardized protocol (10). The middle cerebral artery (MCA), posterior cerebral artery (PCA), vertebral arteries (VA), and basilar artery (BA) were identified. Because of the usual anatomic course of the anterior cerebral artery, the TCD assessment of this vessel is difficult, and the accuracy of mean velocity (Vm) measurement is small so we excluded ACA flow parameters from this study (10, 11). Vm and Gosling's pulsatility index (PI) were measured and recorded at a 54–56 mm depth in both MCAs, at 55–65 mm in both PCAs, at 50–70 mm in both VAs, and at 80–100 mm at BA. Gosling's PI was calculated as the difference between Vmax and Vmin, divided by the mean velocity. TCD parameters were measured as instant variables. Each artery measurements were recorded three times with the probe fixed in position, then the values were averaged. Only measurements with the best signal-to-noise ratio were used, and the highest values for CBF velocities were selected for analysis. TCD tests were always performed by the same physician experienced in the field of the neurosonology (MN). Before every TCD examination blood pressure and heart rate were measured. Most of the TCD investigations (75%) were performed on the visit when mAbs were prescribed. The remained TCDs were performed not earlier than 3 months before starting mAbs therapy.

Data were tested for normal distribution with the Shapiro–Wilk test. In the case of the normal distribution, to compare the mean values, the Student's *t*-test for independent variables was used. Non-parametric *U* Mann–Whitney test was used to compare continuous variables between two groups of observations. The chi-square test or the Fisher test was used to test the relationship between categorical variables. The Wilcoxon test for paired observations with Bonferroni correction was used to compare TCD parameters between the sides of the brain. This test examines the significance of the difference in the distributions of two interdependent variables. Paired Student's *t*-test was used for the analysis of two normally distributed dependent variables. To investigate the existence of monotonic relationships between two variables, Spearman's correlation coefficient was used. The statistically significant result concerning Spearman's correlation coefficient proves the existence of monotonic dependencies between the variables, but the statistically significant result concerning the Pearson correlation coefficient proves the existence of linear dependencies between the variables. Exploratory stepwise multiple logistic regression analysis was used for identifying independent predictors of ≥50% response to mAbs treatment. When preparing our multivariate logistic model for identifying independent predictors of MMDs ≥50% response, we considered independent variables, selected from the database, including age, sex (M/F), duration of disease (years), type of migraine (episodic/chronic), presence of aura (yes/no), number of additional migraine symptoms, pulsating type of pain (yes/no), localization of pain, presence of MOH (yes/no), responsiveness to triptan (yes/no), baseline MMD, MHD, AMD, HIT, and NRS, number of prior preventive classes failures, mood disorders (yes/no), thyroid disease (yes/no), oral contraceptives (yes/no), hypertensive signals MRI (yes/no), family history of migraine (yes/no), and TCD parameters (PI, MCA P;PI, MCA L;Vm, MCA P;Vm, MCA L;Vm, BA;PI, BA;Vm, VA;P Vm, VA L;PI VA P; and PI VA L). From those factors (independent variables), an optimal set of parameters was selected to build a regression model. The process of selecting the optimal set of prognostic factors was performed using a backward stepwise regression, starting with the model with all potential prognostic factors and eliminating irrelevant variables in subsequent steps minimalizing Akaike Information Criterion (AIC). As a result of the analysis, 8 parameters were chosen. No adjustments for multiple comparisons were made, as we had only one final model which is not connected to another one and, it was chosen using backward stepwise regression with AIC, not based on the significance level.

In the case of this analysis, the level of statistical significance was set to *p* = 0.05. All calculations were done in *R (version 4.0.2)*.

## Results

We found 148 potentially eligible patients, 14 were lost to follow-up, 5 had inadequate bone windows and poor TCD baseline measurements, and 6 had concomitant disorders and treatment that could interfere with CBF. Finally, 123 patients were enrolled in this study, all patients received the same dose of erenumab (70 mg per month) or fremanezumab (225 mg per month) for at least 3 months, 94.3% were women. The mean age was 38.72 ± 10.48 years (range: 18–70 years). Thirty-six (29.3%) were diagnosed with episodic migraine, while 87 (70.7%) were patients with chronic migraine. Seventeen (13.8%) patients were diagnosed with migraine with aura. MOH was additionally diagnosed in 62 (50.4%) patients. The duration of the disease was 18.6 years (range from 2 and 50 years). A total of 72 (58.5%) patients achieved ≥50% reduction in MMD after the treatment, while 43 (35%) achieved ≥75% reduction in MMD. The mean treatment duration was 8 months. There was a statistically significant decrease in MMDs, MHDs, AMDs, NRS, and HIT scores after the treatment compared with baseline parameters in both groups. Patients were overusing mostly triptans, combination codeine medicines, paracetamol, ketoprofen, or ibuprofen. Good responders were predominantly younger, and had longer duration of disease and unilateral localization of pain as compared with non-responders. There was one allergic reaction that led to discontinuation of the treatment after 3 months, and 3 patients developed mild constipation. The concomitant medications were mostly contraceptives, antidepressants, and levothyroxine, however, there was no significant difference in using them between groups. The characteristics of the patients depending on treatment efficacy are presented in Table 1.

**Table 1:** Clinical characteristics of migraine patients treated with anti-CGRP mAbs depending on treatment efficacy.

**Parameter**	**Good responders ≥50% RR**	**Non-responders <50% RR**	***p*-value**
	***n*** **= 72**	***n*** **= 51**	
Age (mean ± SD)	40.74 (11.04)	35.86 (8.94)	0.008
Sex, *n* (%)			
Woman	69 (95.8)	47 (92.2)	0.4472
Man	3 (4.2)	4 (7.8)	
BMI. kg/m^2^	23.5	22.4	0.241
Duration of disease (years) (mean ± SD)	20.74 (11.03)	15.57 (9.57)	0.01
Type of migraine, *n* (%)			
Episodic	22 (30.6)	14 (27.5)	0.8637
Chronic	50 (69.4)	37 (72.5)	
Migraine with aura, *n* (%)	9 (12.5)	8 (15.7)	0.8109
Additional migraine symptoms *n* (%)			0.822
One	12 (16.7)	8 (15.7)	
Two	24 (33.3)	21 (41.2)	
Three	26 (36.1)	17 (33.3)	
Four	10 (13.9)	5 (9.8)	
Pulsating type of pain *n* (%)	52 (72.2)	37 (72.5)	1
Localization of pain *n* (%)			0.02
Bilateral	11 (15.3)	19 (37.3)	
Unilateral (variable side)	29 (40.3)	15 (29.4)	
Unilateral (fixed side)	32 (44.4)	17 (33.3)	
MOH, *n* (%)	36 (50)	26 (51)	1
Triptan responders *n* (%)	55 (77.5)	29 (59.2)	0.0517
Botulinum toxineBoNT-A responders, *n* (%)			
Effective	4 (40)	4 (44.4)	1
Ineffective	6 (60)	5 (55.6)	
Type of mAbs, *n* (%)			0.0034
Erenumab	35 (48.6)	40 (78.4)	
Fremanezumab	37 (51.4)	11 (21.6)	
Prior preventive classes failures, *n* (%)			0.2608
0	33 (45.8)	26 (51)	
1	13 (18.1)	8 (15.7)	
2	13 (18.1)	3 (5.9)	
3	7 (9.7)	9 (17.6)	
>4	6 (8.3)	5 (9.8)	
Acute medication used/overused, *n* (%)			0.473
Triptan	30 (41.7)	24 (47.1)	
Codeine	14 (19.4)	11 (21.6)	
NLPZ	17 (23.6)	13 (25.5)	
Triptan+codeine	11 (15.3)	3 (5.9)	
MMD—Baseline	11.93 (5.12)	11.31 (5.31)	0.336
MMD—Post-treatment	2.74 (2.17)	9.41 (3.64)	<0.001
MHD—Baseline	19.11 (8.23)	19.61 (8.45)	0.7055
MHD—Post-treatment	4.19 (3.47)	13.94 (5.6)	<0.001
AMD—Baseline	15.53 (8.67)	15.24 (8.2)	0.9753
AMD—Post-treatment	3.15 (2.92)	10.65 (5.15)	<0.001
NRS—Baseline	8.46 (1.31)	8.49 (1.29)	0.9872
NRS—Post-treatment	6.23 (1.12)	7.58 (1.26)	0.012
HIT-6—Baseline	69.31 (5.01)	68.31 (5.17)	0.2679
HIT-6—Post-treatment	45.43 (6.12)	58.67 (5.98)	0.003
Mood disorders, *n* (%)	18 (25)	8 (15.7)	0.3067
Thyroid disease, *n* (%)	12 (16.7)	13 (25.5)	0.3317
Oral contraceptives, *n* (%)	7 (9.7)	5 (9.8)	1
Hyperintense signals MRI, *n* (%)	7 (9.7)	4 (7.8)	1
Family history of migraine, *n* (%)	41 (56.9)	21 (41.2)	0.1235

*RR, responders rate; SD, standard deviation; BMI, body mass index; NRS, numeric rate scale; HIT-6, headache impact test-6; MOH, medication overuse headache; MMD, monthly migraine days; MHD, monthly headache days; AMD, monthly medication days; mAbs, monoclonal antibodies; MRI, magnetic resonance imaging*.

There were no significant differences regarding baseline Vm and PI between right and left hemispheres except for Vm in VA in the GR group and Vm in PCA in the non-R group. Baseline Vm values in both MCAs were significantly lower in good responders compared with non-responders. There were no PI differences between groups in any arteries (Table 2). Blood pressure and heart rate values did not significantly differ between groups.

**Table 2:** The Vm and PI values before starting anti-CGRP mAbs treatment depending on treatment efficacy and brain side.

**Parameters**	**Good responders (≥50% RR)**	**Non-responders (<50%RR)**	***p*** **-value**
	***n*** **= 72**	***n*** **= 51**	
Baseline Vm, MCA R (cm/s)	64.22 (15.17)	73.14 (12.96)	<0.001
Baseline Vm. MCA L (cm/s)	65.78 (13.37)	73.16 (15.62)	0.0073
*p*-value (difference R/L)	0.0943	0.9885	
Baseline PI. MCA P	0.82 (0.11)	0.84 (0.13)	0.4749
Baseline PI, MCA L	0.81 (0.13)	0.82 (0.15)	0.7972
*p*-value	0.4943	0.1699	
Baseline Vm, PCA R (cm/s)	40.07 (7.4)	41.8 (8.33)	0.2383
Baseline Vm, PCA L (cm/s)	41.91 (9.04)	45.29 (10.83)	0.1869
*p*-value	0.0853	0.0164	
Baseline PI, PCA P	0.82 (0.12)	0.81 (0.13)	0.5365
Baseline PI, PCA L	0.81 (0.12)	0.8 (0.13)	0.6093
*p*-value	0.7605	0.487	
Baseline Vm, VA R	39.54 (10.13)	41.96 (10.42)	0.2033
Baseline Vm, VA L	41.91 (9.04)	45.29 (10.83)	0.2685
*p*-value	0.0534	0.0781	
Baseline PI, VA R	0.82 (0.15)	0.82 (0.14)	0.6146
Baseline PI, VA L	0.82 (0.11)	0.8 (0.14)	0.906
*p*-value	0.4826	0.2514	
Baseline Vm, BA	43.7 (10.69)	44.72 (11.01)	0.6109
Baseline PI, BA	0.82 (0.13)	0.82 (0.13)	0.6038

*RR, responders rate; Vm, mean velocity; MCA, middle cerebral artery; PCA, posterior cerebral artery; VA, vertebral artery, BA, basilar artery; R, right; L, left*.

Given the results presented in Tables 1, 2, we decided to go a step further and prepare a multivariate logistic model to evaluate factors connected with the presence of ≥50% response treatment. MAbs responsiveness ≥50% was positively associated with unilateral pain localization (OR: 6.53, 95% CI: 2.01–23.93; *p* = 0.003) and HIT-6 score (OR: 1.14, 95% CI: 1.01–1.30; *p* = 0.036) whereas negatively associated with Vm in right MCA (OR: 0.96, 95% CI:0.92–0.99; *p* = 0.012), and having no relatives with migraine (OR: 0.40, 95% CI: 0.16–0.95; *p* = 0.040) (Table 3).

**Table 3:** Multivariate logistic model evaluating independent variables associated with the presence of ≥50% responsiveness to anti-CGRP mAbs.

**Parameter**	**OR**	**2.5% CI**	**97.5% CI**	***p*** **-value**
**Multivariate logistic regression model**				
Duration of migraine (years)	1.04	1.00	1.09	0.059
Presence of chronic migraine	0.30	0.08	1.06	0.067
Baseline HIT-6 scale score	1.14	1.01	1.30	0.036
Localization of pain (unilateral variable)	6.53	2.01	23.93	0.003
Localization of pain (unilateral fixed)	6.95	2.21	24.46	0.001
No relatives with migraine	0.40	0.16	0.95	0.040
Vm, MCA R (cm/s)	0.96	0.92	0.99	0.012
Vm, BA	1.04	0.99	1.10	0.107

*HIT-6, headache impact test-6; Vm, mean velocity; MCA, middle cerebral artery; BA, basilar artery; R, right*.

We also found a positive, significant correlation between Vm in both MCAs and post-treatment MMD, MHD, and AMD, a positive, significant correlation between Vm in right MCA and BA and baseline NRS.

## Discussion

In this study, we partly confirmed the findings from our previous pilot study that mAbs good responders had significantly lower baseline Vm in selected brain arteries as compared to non-responders. The pilot study was a small prospective trial that included migraineurs treated with erenumab and evaluated clinically and by means of TCD before and after treatment. We noticed that baseline Vm values in MCA R, VA R and BA were significantly reduced in good responders (≥50% reduction in MMD) as compared with the non-responders group (8). Thus, we concluded that lower baseline Vm in right CA may predict erenumab efficacy. The present study was conducted to check if these results could be replicated in a larger cohort. Indeed, we confirmed that the Vm value was significantly diminished not only in the right but also in the left MCA in good responders, while we failed to find a difference regarding the other arteries between groups. We also found that Vm in the right MCA might predict Mabs efficacy.

Therefore, the question arises of how to explain this baseline flow velocity difference between good and non-responders? To proceed further it should be explained that brain arteries differ from arteries localized in other parts of the body, as have their own muscle tone, respond with an active contraction to the increase in transmural pressure, and stay in partial contraction, which allows them to change their diameter to regulate CBF. Blood flow is related to the diameter of the vessel, thus even a slight increase in the vessel's muscle tone will reduce its diameter and disturb the flow (12, 13). Assuming that the growing diameter of the artery reduces blood flow velocity, lower Vm in GR may also indicate more dilated arteries in this group as compared to non-responders. Lassen et al. revealed that CGRP infusion dilated the MCA in patients with migraine, they observed a 7.5% increase in MCA diameter corresponding to a 17% increase in its cross-sectional area. They concluded that CGRP can cross the blood–brain barrier to some extent in the large human cerebral arteries (5). Contrary to this observation, Asghar et al. noticed that GCRP causes dilation of the middle meningeal artery but not the MCA in healthy volunteers (7). As CGRP is a powerful vasodilator, one possible explanation of our findings would be that GR has increased interictal secretion of endogenous perivascular CGRP than non-responders. Indeed, differences in salivary levels of CGRP between patients and controls were found not only during migraine attacks but also in the interictal phase (14, 15). Interestingly, Greco et al. assessed CM patients with overuse of medications and found that responders to the detoxification had significantly higher baseline levels of CGRP as compared to non-responders (16). They found CGRP level as a potential peripheral marker associated with migraine subtypes and disease severity.

Assuming that the diameter of the artery does not change, velocity correlates with CBF—therefore an increase in CBF increases blood flow velocity. Thus, in our study, one of the possible explanations for reduced Vm in MCAs of good responders could be reduced CBF in anterior circulation in this group. So far there are no consistent data regarding CBF in migraineurs as compared to controls, and very scarce data about it between different migraine groups. Generally, findings from the meta-analyses confirm that migraineurs have altered cerebrovascular function, especially higher resting mead blood flow velocity (MBFV), higher PI and lower cerebrovascular resistance (CVR) to hypercapnia in the posterior circulation and higher resting MBFV in the anterior circulation. Moreover, there are differences between those parameters between migraineurs with and without aura (17). A SPECT study revealed that the headache scores in migraineurs were significantly correlated to the regional CBF (rCBF) on the bilateral temporal lobes and right frontal lobe. The migraineurs had lower rCBF at the frontal and temporal lobes, and the lower rCBF was correlated to the degree of headache (18). In a very interesting TCD study Lee et al. assessed longitudinal changes in CBF velocities according to the clinical course of migraine. In the remission group, a decrease in CBF in MCA and BA was found after 2 years of observation. Contrary, the progression group showed increasing CBF in the bilateral MCAs. In patients with the persistence course CBF generally remained unchanged (19). Interestingly, the baseline Vm also differed between groups (19). The authors concluded that migraine patients may have intra-individual variations in CBF and that longitudinal changes in CBF might be associated with the clinical course of migraine.

Another explanation of the Vm difference between good and no-responders may be the age difference, as in our study GR were about 5 years older than non-responders. Flow velocities in basal cerebral arteries range widely and are significantly age-related, namely blood flow velocities decrease in all vessels with advancing age. Contrary, PI increases with age (20, 21). Those changes reflect arteriosclerosis and arterial stiffness. Thus, in GR lower Vm in MCAs might be linked with older age. Nevertheless, the theory that Vm difference between groups may have resulted from a young age can be excluded, first, because Vm has been lower only in MCAs and did not differ in other arteries, and second, because there was no difference regarding PI between groups (and with decreasing Vm, PI should increase). The second theory explaining the Vm difference between groups may be linked with triptans use. It is reported that CBF decreases significantly after triptans administration due to vasoconstriction (22). Thus, it is possible, that patients overusing triptans may have different Vm compared with other migraineurs and this would explain our study findings. However, we did not find any difference regarding the presence of MOH or triptan use between GR and non-R in our study.

Another aim of our study was to identify clinical predictors of responsiveness to mAbs. Based on our statistic model, unilateral localization of pain and HIT-6 scale were positively associated with mAbs responsiveness ≥50% of MMD, while no relatives with migraine and Vm in right MCA were negatively associated. Our results partly confirm findings from a study by Brabanti et al. who tried to evaluate factors that influence the outcome in CM and high-frequency migraine (HFEM) in patients treated with erenumab. Treatment responsiveness in HFEM was positively associated with unilateral pain localization (OR: 3.03, 95% CI: 1.24–7.40; *p* = 0.015), whereas in CM responsiveness was positively associated with and baseline migraine frequency (OR: 1.06, 95% CI: 1.02–1.11; *p* = 0.031), dopaminergic symptoms (OR: 2.01, 95% CI: 1.14–3.52; *p* = 0.015), and negatively associated with psychiatric comorbidities (23). Similarly, unilateral pain, good response to triptans, and normal weight, was linked with a good response to galcanezumab in CM patients (24). In a study by Iannote et al. fewer migraine days at baseline were associated with ≥ 50% response rate at 1 month and fewer MMDs, years of chronic migraine, and monthly analgesic use at 6 months (25). Another study revealed that age at migraine onset, number of failed preventive medications, and MIDAS score were associated with >75% erenumab response (26). Although several authors reported a link between response to triptans and response to erenumab, we did not find a similar significant association in our study (27, 28).

Not only responsiveness to Mabs in migraine seems to be related to pain localization. Interestingly, unilateral pain was associated with being pain-free at 2 h after triptan use and also correlated with good outcomes in CM patients treated with onabotulinumtoxin A (29, 30). According to several authors, the presence of unilateral pain indicates a “pure” migraine, with less tensional component and therefore with a higher likelihood of being good responder (30). Unilateral pain in CM patients may be connected with peripheral trigeminal sensitization also during the chronic phase (30). The question arises if having relatives with migraine may be also a factor linked with “pure” migraine, thus positively associate with mAbs responsiveness? Guo et al. found no statistical association between familial aggregation of migraine and hypersensitivity to CGRP infusion in migraine without aura patients (31). On the other hand, specific clinical features of migraine, like lower age-at-onset, a higher number of medication days, and migraine with aura seem more determined by genetic factors (32).

It should be emphasized that our cohort may differ from studies groups from other countries. First, as in Poland there is no reimbursement of mAbs for migraine patients, the migraineurs cover the cost of treatment by themselves, using discounts programs provided by Novartis and Teva (where the price is reduced up to 50%) and in the case of lack of treatment effectiveness, they usually give up treatment earlier than recommended. To our best knowledge, our study is the first study of anty-CGRP mAbs in migraine prophylaxis in the Polish population (except from our previous pilot study), one of the very few from this part of Europe, and the first study of a population with no treatment reimbursement.

The limitation of our study may be the time point for the efficacy evaluation, as we could miss non-responders who, from economic reasons, decided to stop treatment earlier. Besides, it should be remembered that TCD measures only the flow velocity and not the absolute CBF value. The correlation between CBF and flow velocity is variable. Cerebral blood velocity is an adequate surrogate of absolute flow only if the insonated vessel maintains constant vessel diameter across time and experimental conditions. Blood flow velocity is further influenced by several factors, including arterial blood pressure, ICP, hematocrit, PaCO_2_, and the status of autoregulation, thus making a direct comparison of flow velocity and CBF difficult. Another limitation of any CBF in migraine study is that in high frequent episodic or chronic migraine, even when the examinations are performed outside the headache phase, it cannot be excluded that patients are in the early prodromal or late postictal phases. The other limitation is that we did not exclude patients with a non-migraine headache on the day of TCD assessment (due to a large percentage of CM patients included in our study). Moreover, the next limitation may be the age difference between good responders and non-responders' groups, as well as pain localization and duration of disease difference between groups.

## Conclusions

This is the first real-life Polish study that confirms that mAbs are highly effective and tolerated in both EM and CM. Baseline Vm in both MCAs is lower in mAbs good responders as compared with non-responders which may reflect increased vasodilation or decreased CBF in this group. Increased interictal secretion of CGRP in good responders may explain the study findings. Future research should try to find out if the baseline CGRP serum level may predict mAbs efficacy. Our study suggests that mAbs responsiveness is positively associated with unilateral pain localization and headache burden and negatively associated with right Vm and lack of migraine family history.

## Data Availability Statement

The original contributions presented in the study are included in the article/supplementary material, further inquiries can be directed to the corresponding author/s.

## Ethics Statement

The studies involving human participants were reviewed and approved by Local Ethics Committee of the Ludwik Rydygier Collegium Medicum in Bydgoszcz. Written informed consent for participation was not required for this study in accordance with the national legislation and the institutional requirements.

## Author Contributions

MN conceptualization of the study, acquisition of data, analysis and interpretation of the data, and drafting of the manuscript. WK and MS revising the manuscript for intellectual content. JJ-K acquisition of data and revising the manuscript for intellectual content. GM and MW-P acquisition of data. All authors contributed to the article and approved the submitted version.

## Conflict of Interest

MN and MS have received lecture honoraria from Novartis Poland Sp. z o.o. and Teva Pharmaceuticals Polska Sp. z o.o. The remaining authors declare that the research was conducted in the absence of any commercial or financial relationships that could be construed as a potential conflict of interest.

## Publisher's Note

All claims expressed in this article are solely those of the authors and do not necessarily represent those of their affiliated organizations, or those of the publisher, the editors and the reviewers. Any product that may be evaluated in this article, or claim that may be made by its manufacturer, is not guaranteed or endorsed by the publisher.
